# The relationship between malnutrition risk and inflammatory biomarkers in outpatient geriatric population

**DOI:** 10.1007/s41999-020-00303-4

**Published:** 2020-03-06

**Authors:** Paulina Fatyga, Agnieszka Pac, Małgorzata Fedyk-Łukasik, Tomasz Grodzicki, Anna Skalska

**Affiliations:** 1 Department of Toxicology and Environmental Diseases, Jagiellonian University Medical College, Kraków, Poland; 2 Department of Internal Medicine and Gerontology, Jagiellonian University Medical College, 2 Jakubowskiego Str., 30-688 Kraków, Poland; 3 Chair of Epidemiology and Preventive Medicine, Jagiellonian University Medical College, Kraków, Poland; 4 University Hospital, Kraków, Poland

**Keywords:** Malnutrition risk, MNA, Older persons, Inflammatory biomarkers

## Abstract

**Purpose:**

Malnutrition is an underestimated, but significant problem among older persons. It is described as a consequence of genetic and environmental factors, lack of physical activity, and co-morbidities. However, a key role of a geriatrician is to further explore the multidimensional complexity of this issue. The aim of this study was to identify the relationship between nutritional status and different factors, particularly focusing on inflammatory biomarkers.

**Methods:**

Nutritional status was assessed using Mini-Nutritional-Assessment with a score below 24 (out of 30) defined as malnutrition. Different serum biomarkers of inflammation were measured, such as High-Sensitivity-C-Reactive-Protein (hsCRP), Interleukin-6 (IL-6), Interleukin-8 (IL-8), Interleukin-18(IL-18), osteoprotegerin(OPG), and Soluble-Receptor-For-TNF-alfa(sTNFRII). Medical history, mental status (Mini-Mental-State-Examination, Geriatric-Depression-Scale) and activities of daily living (using Instrumental-Activities-of-Daily-Living-Scale) were used in the evaluation. The relationship between nutritional status and the factors listed was assessed.

**Results:**

The mean age of 76 examined persons (40.8% female) from the outpatient clinic was 71 years. Malnutrition risk was recognized in 29%. The following factors significant in univariate regression were used in stepwise regression analysis: age, sex, mental status (MMSE, GDS), valve disease, number of diseases, IADL. Stepwise regression revealed that the risk of malnutrition was increased by the presence of valve disease, number of diseases, and female sex. Factors that increased the risk of malnutrition were: logsTNFRII (OR = 3.09; 95% CI 1.07–8.96), IL-8 (OR = 1.09; 95% CI 1.00–1.18), and OPG (OR = 1.27; 95% CI 1.02–1.57). Risk of malnutrition was negatively associated with Il-18(OR = 0.995; 95% CI 0.991–0.999).

**Conclusions:**

Chronic inflammation and immunologic process are likely contributors to the complex etiopathogenesis of malnutrition in older persons.

## Key summary points

**Aim:**

It is crucial to identify factors contributing to malnutrition risk in older persons in order to prevent malnutrition as far as possible.

**Findings:**

Factors that increased the risk of malnutrition were: increased levels of IL-8, osteoprotegerin (OPG), and Soluble-Receptor-For-TNF-alfa (sTNFRII; log transformed). In comparison with previous studies, in our study there was no significant difference in hsCRP and IL-6 in participants at risk of malnutrition and those who were well-nourished, nevertheless, those at risk of malnutrition had significantly higher IL-8, OPG and sTNFRII concentrations, but higher levels of IL-18.

**Message:**

The etiopathogenesis of malnutrition in older persons is complex and our study indicated that chronic inflammation plays a probable role and should be considered in evaluating nutritional status in the geriatric population; however, it also exposes an avenue where further research is needed in order to enhance our understanding and guide comprehensive patient care.

## Introduction

Nutrition plays a pivotal role in life. Although the vast majority of nutrition-focused efforts are dedicated to the epidemic of obesity, on the other end of the spectrum, malnutrition is a significant problem posing increasing concern in our geriatric population. The term “malnutrition” is used to describe a host of nutritional abnormalities. Typically, it refers to protein–energy malnutrition that occurs when metabolic requirements chronically exceed nutritional intake, resulting in a longstanding negative balance of both energy and protein [1]. According to the Global Leadership Initiative on Malnutrition (GLIM) that was convened in January 2019, for establishing the diagnosis of malnutrition the combination of at least one phenotypic and one etiologic criterion is required [2]. To be more detailed, phenotypic criteria are as follows: non-volitional weight loss, low body mass index, reduced muscle mass. In addition to this, etiologic criteria include reduced food intake or assimilation and disease burden/inflammatory condition. Nevertheless, the recommended GLIM approach uses only phenotypic criteria cut-points to provide for severity grading [2].

Severe malnutrition leads to cachexia, a complex metabolic syndrome associated with underlying illness and inflammation which contributes to increased catabolism and increased muscle protein breakdown. It is characterized by loss of muscle with or without loss of fat mass. The prominent clinical feature of cachexia is weight loss in adults (corrected for fluid retention) or growth failure in children (excluding endocrine disorders) [3]. Cachexia is usually associated with severe illness and the rate of change in the form of weight loss and muscle wasting occurs much more rapidly than in age-related and disease-related muscle loss [4].

It is recommended that the GLIM consensus criteria should be applied to diagnose malnutrition in persons with sarcopenia, cachexia and frailty so that the priority to undertake appropriate nutrition interventions may be recognized.

The important issue is that malnutrition is a risk factor for increased morbidity and decreased quality of life [5–7]. Its presence extends the duration of treatment and hospitalization, increases the cost of treatment, the risk of disability and dependency, as well as mortality [8–11]. Many factors contribute to the occurrence of malnutrition including co- and multi-morbidity, medications used, disability, and psychosocial factors; including loneliness, poverty, and the aging process itself. The aging process is accompanied by physiologic changes leading to the reduction of appetite, gastrointestinal motility disorders, decreased level of anabolic hormones, and dysregulation of the immune system. The effect of these changes is the increase in body weight in early old age associated with a decrease in lean body mass and simultaneous increase in body fat. After the stabilization period, a weight loss of 1–2 kg is observed in each subsequent decade, with a reduction in the weight of both tissues in late old age. Inflammaging, a state of chronic moderate inflammation, is a factor that may contribute to loss of appetite, inhibition of albumin production, and weight loss. It is characterized by increased levels of pro-inflammatory cytokines, especially interleukin 6 (IL-6) and tumor necrosis factor-alfa (TNFα).

Increasing evidence from basic biological studies suggests that inflammatory cytokines play a direct role in the development of typical manifestation of aging, such as sarcopenia, anaemia, and cognitive decline. Higher levels of pro-inflammatory cytokines were found in the majority of older people, also in the absence of clinically active diseases [12] and without signs of infections [13]. Moreover, they are associated with clinical progression of chronic disease states. Increased levels of high sensitive C-reactive protein (hsCRP), TNFα and its soluble receptors, IL-6, IL-1, IL-8, IL-18 were found in different chronic conditions such as atherosclerosis, cardiovascular disease, chronic heart failure (CHF), diabetes, chronic obstructive pulmonary disease, chronic kidney disease, and cancer. Also osteoprotegerin (OPG), a member of TNFα receptor superfamily turned out to be a predictor of complications, cardiovascular risk, and of poor prognosis in many chronic conditions [14–17]. Increased level of OPG was predictive of hospitalization for heart failure in patients with advanced systolic CHF and ischemic heart disease independently of conventional risk markers [16].

Nonetheless, there is an evidence of a relationship between malnutrition and inflammation expressed by elevated levels of pro-inflammatory cytokines and these biomarkers were independent risk factors of malnutrition in various disease states, such as renal failure [18], neoplastic disease [19], and Alzheimer's disease [20].

Many laboratory indicators are used to assess malnutrition and its risk. Abd-Elraheem et al*.* stated that among different factors like serum levels of albumin, transferrin, total cholesterol, vitamin D, and lymphocyte count, low serum albumin levels best predict 1-year mortality in hospitalized older adults, followed by low transferrin serum levels [21]. But described independent relationships between malnutrition and inflammatory markers indicate that there exists a need for further investigation of the link between malnutrition and inflammation in old age.

For that reason the aim of this study was to identify the relationship between nutritional status and different factors, particularly focusing on inflammatory biomarkers in older patients.

Because pro-inflammatory cytokines are involved in both, ageing and chronic conditions, from among many inflammatory markers we decided to assess those explored earlier (hsCRP, IL-6), and those involved in the development of chronic diseases and its complications, especially cardiovascular diseases often occurring in older people, but less frequently studied in the context of malnutrition, like OPG, IL-18, soluble receptor II for TNFα (sTNFRII).

## Patients and methods

### Study design and patient enrollment

A cross-sectional study design was utilized. Patients aged 60 years or older followed in the Geriatric Outpatient Clinic were included from October 2010 to February 2014. All individuals signed an informed consent to participate in the study.

The most significant exclusion criterion was active inflammatory state or exacerbation of chronic inflammation/chronic condition, another exclusion criteria were: immobility, Mini-Mental State Examination (MMSE) score below 10 and if the patient had dementia or cognitive impairment, the interview was completed by a proxy. It is worth noting that all participants came to the clinic for routine checks independently in the stable period of the disease. In all patients, demographic data, smoking status, past/current medical history, and medication history were obtained using a structured questionnaire. The obtained data from questionnaires was supplemented with information from medical records. Weight (kg) and height (m) were measured and BMI (kg/m^2^) was calculated according to the formula: weight (kg)/(height (m))^2^.

Cognitive function was assessed using the MMSE, in which a score below 24 of 30 suggested cognitive impairment. Mood was assessed with the 30-point Geriatric Depression Scale (GDS), in which a score of 11 points and higher suggested depression.

The risk of malnutrition was evaluated using the Mini Nutritional Assessment (MNA) with a maximum score of 30 points; 24 points and higher suggested normal nutritional status, 17–23.5 points suggested an increased risk of malnutrition, and below 17 points suggested malnutrition. In our sample, the minimum MNA score was 18.5; therefore, the MNA score of less than 24 was used as a categorical variable.

Functional status was assessed by the Instrumental Activities of Daily Living (IADL) scale with a maximum score of 27 points. The higher the score, the better the functional status.

Handgrip strength was measured with hydraulic hand dynamometer Model SH5001, SEAHEN Corp. MASAN Korea.

Muscle mass including total lean body mass (LBM), and appendicular lean mass (aLM) was examined using a dual-energy X-ray absorptiometry (DXA) (Lunar Prodigy, General Electric Medical Systems).

Human interleukin immunoassay kits were used to assess serum IL-6 and interleukin-8 (IL-8) levels including Human IL-6 immunoassay Quantikine^®^ HS ELISA and Human CXCL8/IL-8 Immunoassay Quantikine^®^ ELISA (R&D Systems, Inc., 614 McKinley Place NE, Minneapolis, MN 55413, USA). Serum IL-18 level was measured by Human IL-18 ELISA Kit (Medical & Biological Laboratories CO., Japan). For the quantitative measurement of sTNFRII serum concentrations, Human TNFRII/TNFRSF1B Immunoassay Quantikine^®^ ELISA (R&D Systems, Inc. 614 McKinley Place NE, Minneapolis, MN 55413, USA) was used. Osteoprotegerin (OPG) concentration was determined using sandwich enzyme immunoassay, the RD194003200 Human Osteoprotegerin ELISA kit (BioVendor – Laboratorni medicina a.s. Karasek 1767/1, 621 00 Brno, Czech Republic).

The study was approved by the Bioethical Committee of Jagiellonian University (Kraków, Poland).

### Statistical analysis

Due to the relatively small sample size of the study subgroups as well as the non-normal distribution of variables, the descriptive characteristics were presented as medians and interquartile range (IQR) for continuous variables and number and relative frequency for nominal data. The two study groups, “risk of malnutrition” and “no risk of malnutrition”, were compared with *U* Mann–Whitney test and $\chi^{2}$ test, respectively. The relationship between qualitative variables was assessed by Spearman’s rank correlation coefficient. Binary logistic stepwise regression model, with the risk of malnutrition as the dependent variable, was used to assess the characteristics related to the risk of malnutrition. The initial set of independent variables used in each model was as follows: age, sex, valve disease, number of diseases, GDS, MMSE, IADL, and inflammatory biomarkers. The *p* value was set at 0.05 for entering variables in the logistic regression model in a stepwise procedure. The results were expressed as Odds Ratio (OR) together with 95% confidence intervals (95% CI). The statistical analysis was carried out in STATA/SE 14 (Stata Corp, College Station, Texas, United States). In all analyses, 2-sided tests were used. *p* values < 0.05 were considered significant.

## Results

Mean age (IQR of 76 participants; 40.8% female) was 71 years (67; 76.5). The most common disease was hypertension followed by coronary heart disease (CHD), chronic heart failure (CHF), osteoarthrosis, history of myocardial infarction (MI), and diabetes. A quarter of the patients were diagnosed with valve disease, mainly mitral insufficiency developed in the course of CHD and heart failure. Those, with valve disease had more advanced heart failure in the form of a lower ejection fraction (data not shown). We divided the examined persons into two groups: the group without the risk of malnutrition (according to the MNA score ≥ 24) and the group at risk of malnutrition (with MNA score < 24). These groups did not differ in terms of age, sex, smoking history, BMI, number of diseases, number of medications, the presence of diseases (i.e. CHF, CHD, MI, stroke, diabetes mellitus, hypertension, osteoarthrosis), MMSE score, GDS score, or IADL score. Furthermore, there was no significant difference in muscle mass between well-nourished and patients at risk of malnutrition. Nevertheless, the groups varied by the presence of valve disease (Table 1).

**Table 1:** The characteristics of study group

Variable	All (*n* = 76)	MNA ≥ 24 (*n* = 54)	MNA < 24 (*n* = 22)	*p* value
Age (years) (median; IQR)	71 [67; 76.5]	70.5 [67; 76]	71.5 [69; 77]	0.17
Sex (female; %)	40.8	35.2	54.6	0.12
Smokers (%)	11.8	12.9	9.1	0.63
Pack-years (median; IQR)	0 [0; 25]	0 [0; 25]	4.25 [0; 22.5]	0.78
BMI (kg/m^2^; median; IQR)	28.1 [25.9; 30.9]	28.7 [26.4; 31.3]	26.53 [24.8; 29.7]	0.06
Number of diseases (median; IQR)	5 [4; 7]	5 [4; 6]	6 [4; 7]	0.06
Number of medications (median; IQR)	7 [6; 9]	7 [6; 9]	7 [6; 9]	0.87
CHF (%)	69.7	66.7	77.3	0.36
CHD (%)	73.7	74.1	72.3	0.90
MI (%)	46.1	46.3	45.5	0.95
Stroke (%)	7.9	7.4	9.1	0.80
Diabetes (%)	39.5	35.2	50.0	0.23
Hypertension (%)	89.5	92.6	81.8	0.16
Valve disease (%)	25.0	16.7	45.5	0.01
Osteoarthrosis (%)	59.2	54.0	22.0	0.98
MMSE (median; IQR)	28 [25; 29]	28 [25; 29]	26.5 [25; 28]	0.10
GDS (median; IQR)	8 [6; 14]	8 [6; 14]	9 [7; 16]	0.21
Handgrip strength (kg) (median; IQR)	20 [12; 28]	20 [12.6; 28]	15.3 [10; 22]	0.04
Lean body mass (kg)	49.2 [40.8; 54.1]	49.4 [42.2; 54.2]	44.9 [39.0; 54.0]	0.26
Appendicular muscle mass (kg)	21.1 [16.4; 23.9]	21.2 [17; 23.5]	18.3 [15.8; 24.3]	0.46
aLM/BMI	0.74 [0.58; 0.81]	0.75 [0.58; 0.81]	0.70 [0.61; 0.84]	0.95

*BMI* body mass index, *CHF* chronic heart failure, *CHD* coronary heart disease, *GDS* Geriatric Depression Scale, *IADL* Instrumental Activities of Daily Living, *IQR* interquartile range, *MI* myocardial infarction, *MMSE* Mini-Mental State Examination

Laboratory results were significant for a lower level of hemoglobin in the group at risk for malnutrition. There was no significant difference in albumin and creatinine concentration between the two groups. There was also a significant difference in levels of OPG and sTNFRII between the two study groups, with higher levels found in the group at risk for malnutrition. There was no significant difference in hsCRP, IL-6, IL-8, and IL-18 levels (Table 2).

**Table 2:** The laboratory tests result in the study group, the whole and according to nutrition status

Variable	All (*n* = 76)	MNA ≥ 24 (*n* = 54)	MNA < 24 (*n* = 22)	*P*
Creatinine (µmol/L; median; IQR)	86.1 [62.7; 102.5]	85.8 [68; 102.9]	88.45 [66.4; 102]	0.62
Hemoglobin (g/dL; median; IQR)	13.7 [12.9; 14.7]	13.9 [13.2; 15]	12.9 [12.3; 13.3]	< 0.001
Albumin (g/L; median; IQR)	43.0 [41; 45]	43 [41; 45]	42 [40; 44]	0.07
hsCRP (mg/L; median; IQR)	1.65 [0.77; 3.56]	1.76 [0.79; 3.29]	1.46 [0.57; 6.53]	0.99
IL-6 (pg/ml; median; IQR)	3.61 [2.33; 5.46]	3.61 [2.31; 5.37]	3.51 [2.68; 6.28]	0.57
Osteoprotegerin (pmol/l; median; IQR)	7.53 [6.2; 10.1]	7.22 [6.13; 8.97]	9.52 [6.4; 11.37]	0.04
IL-8 (pg/ml; median; IQR)	11.61 [7.82; 19.68]	11.03 [7.83; 15.98]	13.97 [7.82; 23.48]	0.42
IL-18 (pg/ml; median; IQR)	414.7 [301.5; 507.6]	437.5 [316.7; 556.8]	360.8 [257.4; 455.7]	0.08
sTNFRII (pg/ml; median)	3102 [1978.5; 3901]	2781 [1659; 3701]	3474.5 [2791; 4176]	0.048

*hsCRP *high sensitive C reactive protein, *IL-6* interleukin 6, *IL-8* interleukin 8, *IL-18* interleukin 18, *IQR* interquartile range, *sTNFRII* soluble tumor necrosis factor-α receptor type II

Bearing in mind the relationship between chronic diseases and inflammation, we assessed the relationship between inflammatory markers and possible measurable indicators of disease advancement, which might contribute to malnutrition. We found negative correlations between estimated glomerular filtration rate (eGFR) calculated according to Modification of Diet in Renal Disease (MDRD) and IL-6 ($\rho$ = − 0.39, *p* = 0.006), and sTNFRII ($\rho$ = − 0.61, *p* < 0.0001), positive correlation between IL-18 and creatinine level ($\rho$ = 0.36, *p* = 0.01), and OPG ($\rho$ = 0.34, *p* = 0.02), but no patient had severe kidney failure. Association also was found between inflammatory bio-markers and indicator of heart failure –NT-proBNP. NT-proBNP positively correlated with IL-6 ($\rho$ = 0.40, *p* = 0.005), hsCRP ($\rho$ = 0.30, *p* = 0.04), OPG ($\rho$ = 0.45, *p* = 0.002), and with sTNFRII ($\rho$ = 0.42, *p* = 0.003). Negative correlations were also shown between high-density cholesterol (HDL) and IL-6 ($\rho$ = − 0.30, *p* = 0.03), IL-18 ($\rho$ = − 0.53, *p* = 0.0001), hsCRP ($\rho$ = − 0.34, *p* = 0.02), OPG ($\rho$ = − 0.35, *p* 0.02), and sTNFRII ($\rho$ = − 0.33, *p* = 0.02). Importantly, in assessing the risk of malnutrition, a negative correlation was found between albumin and IL-6 ($\rho$ = − 0.48, *p* = 0.0005) and hsCRP ($\rho$ = − 0.32, *p* = 0.03). Despite the correlations shown, that may indicate a relationship between inflammation and disease progression (chronic kidney disease, heart failure, dyslipidemia), none of these diseases has been associated with the risk of malnutrition in a univariate regression analysis. Only valve disease that may indicate an advanced form of heart failure was significantly associated with the risk of malnutrition in a univariate analysis, which is why its presence was included in the final regression models.

In the context of assessing risk of malnutrition and potential contribution of advanced heart failure it is worth emphasizing that patients with heart failure had higher total muscle mass (49.04 kg vs 41.77 kg, *p* < 0.001) and appendicular muscle mass standardized for height (7.48 kg/m^2^ vs 6.92 kg/m^2^, *p* = 0.03) as well as greater muscle strength (19 [12.35; 29] kg vs 12 [9;17] kg, *p* < 0.001] compared to people without heart failure, which may indicate a lack of cardiac cachexia in HF patients and suggest the contribution of other factors to the risk of malnutrition.

In a stepwise regression analysis, malnutrition served as the dependent variable. A set of potentially contributing factors (significant in a univariate regression analysis) and inflammatory biomarkers were separately introduced to the models. The analysis revealed that the risk of malnutrition increased with a higher number of comorbidities, female sex, cognitive impairment, and inflammation measured by a higher concentration of IL-8, logsTNFRII, and OPG; the risk of malnutrition was negatively associated with Il-18 level (Tables 3, 4, 5, and 6).

**Table 3:** The stepwise logistic regression analysis with IL-8 level as the independent variable

Malnutrition risk	Odds ratio	95% CI	*P*
Valve disease	9.14	1.55; 53.82	0.01
Sex (Male)	0.04	0.01; 0.36	0.004
Number of diseases	1.74	1.09; 2.77	0.02
IL-8 (pg/ml; median; IQR)	1.09	1.00; 1.18	0.04
MMSE (points)	0.76	0.59; 0.98	0.03

**Table 4:** The stepwise logistic regression analysis with logsTNFRII as the independent variable

Malnutrition risk	Odds ratio	95% CI	*P*
Valve disease	4.80	1.23; 18.82	0.02
Sex (Male)	0.13	0.03; 0.55	0.01
Number of diseases	1.47	1.07; 2.01	0.02
logsTNFRII (median; IQR)	3.09	1.07; 8.96	0.04

**Table 5:** The stepwise logistic regression analysis with osteoprotegerin as the independent variable

Malnutrition risk	Odds ratio	95% CI	*P*
Valve disease	4.24	1.12; 16.11	0.03
Sex (Male)	0.12	0.03; 0.53	0.005
Number of diseases	1.44	1.06; 1.95	0.002
OPG (pmol/l; median; IQR)	1.27	1.02; 1.57	0.03

**Table 6:** The stepwise logistic regression analysis with IL-18 as the independent variable

Malnutrition risk	Odds ratio	95% CI	*P*
Valve disease	7.56	1.73; 33.0	0.01
Sex (Male)	0.19	0.05; 0.80	0.02
Number of diseases	1.54	1.12; 2.12	0.01
IL-18 (pg/ml; median; IQR)	0.995	0.991; 0.999	0.047
MMSE (points.)	0.84	0.69; 1.03	0.09

Based on the logistic regression results, higher levels of OPG, IL-8, and sTNFRII were independent predictors of malnutrition risk. Increase of OPG by one [pmol/l] resulted in OR = 1.27 (95% CI 1.022–1.57); increase of IL-8 by one [pg/ml] resulted in OR = 1.09 (95% CI 1.003–1.18); odds of malnutrition increased more than three-fold per each logsTNFRII (OR = 3.09 95% CI 1.07–8.96). An inverse relationship was observed between IL-18 level and malnutrition risk; per increase of IL-18 by one [pg/ml], odds of malnutrition decreased by 0.5% (95% CI 0.991–0.999).

## Discussion

In our study population, the risk of malnutrition was associated with female sex, presence of valve disease, and multiple co-morbidities. Factors that increased the risk of malnutrition were: increased levels of IL-8, OPG, and sTNFRII (log-transformed). The risk of malnutrition was negatively associated with Il-18 level. The obtained results indicate that irrespective of the etiology (inflammaging or related to chronic conditions), inflammation is associated with a greater risk of malnutrition.

Although chronic diseases may lead to cachexia most of our patients were not malnourished, but at risk of malnutrition. Because of a cross-sectional design of this study, we were not able to assess the potential loss of weight, but even in the group at risk of malnutrition median BMI was over 26 kg/m^2^ and both groups did not differ significantly in muscle mass.

Previous studies have demonstrated a relationship between malnutrition and elevated levels of IL-6 and TNFα [22, 23]. It is also known that high level of cytokines TNFα, IL-6, and IL-2 are associated with a decrease in food intake and may contribute to anorexia. Other studies have demonstrated the relationship between malnutrition and inflammatory markers in specific population groups, including in children [24, 25], dialysis patients [14, 15, 18, 26–30], patients with neoplasms [19, 31, 32], and patients with autoimmune diseases [33, 34].

Our study included an expanded panel of inflammatory biomarkers and assessed their association with risk of malnutrition in a population of geriatric counseling patients. Oe et al. also studied a population of older persons, but located in Japan. Their study showed that TNFα was associated with inflammation and insulin resistance in both Japanese older men and women; a prominent association was observed between TNFα and malnutrition status in older women [35]. In another study, Singh et al*.* proposed that inflammation, especially expressed by elevated IL-6 levels, may be a common cause of multiple age-related diseases or a final common pathway by which disease leads to disability and adverse outcomes in older adults [36].

In a study of patients with end-stage renal disease, it was suggested that malnutrition was best predicted by hsCRP and IL-6 levels [18]. In our study, there was no significant difference in hsCRP and IL-6 in participants at risk of malnutrition and those who were well-nourished; however negative correlations between hsCRP and IL-6 levels and albumin concentration were found, and those at risk of malnutrition had significantly higher IL-8 concentrations.

IL-8 is a small protein chemoattractant that recruits neutrophils to sites of inflammation through interaction with at least two types of receptors-A and B [37]. The level of IL-8 is important in evaluating several chronic diseases, such as diabetes, cancer, obesity, and autoimmune disease. Gioulbasanis et al. showed that baseline IL-8 levels correlate with the nutritional status of patients with metastatic non-small-cell lung cancer patients (NSCLC), suggesting that this cytokine may be related to cachexia [19].

Additionally, Abo-Shousha et al. demonstrated that IL-6 and IL-8 levels were higher in children with protein-energy malnutrition than in properly nourished children [25]. Sugawara et al. presented that there is a significant increase in the levels of IL-6 and IL-8 in malnourished patients with chronic obstructive pulmonary disease (COPD). The levels of elevated inflammatory cytokines decreased significantly post nutritional intervention combined with the low-intensity exercise [38].

Yoo et al*.* studied patients with sarcopenia, demonstrating that they had higher levels of CRP and erythrocyte sedimentation rate (ESR) than his control group. Chronic inflammation, associated with increased degradation of muscle protein, was determined to be an important risk factor for hip fracture in sarcopenic patients [39]. In our study group of patients in a stable period of their diseases well-nourished patients and those at risk of malnutrition did not differ in muscle mass.

Further evidence of the relationship of malnutrition with inflammation is the study by Takele et al. who found a significant correlation between low BMI and increased levels of IFN-γ, IL-2, IL-12, IL-4, IL-5, IL-13, IL-10, IL-33, and TNFα; however, not IL-8 or CRP. Their results show that even in the absence of apparent infections, healthy malnourished individuals display dysfunctional immune responses which may cause increased susceptibility to infectious diseases with more severe presentations [40].

As shown above, the presence of chronic diseases can contribute to increase of inflammatory marker levels, but increase of inflammatory cytokines was also observed in malnourished but healthy individuals [25, 40].

Moreover, van der Pols-Vijlbrief et al*.* in their systemic review found moderate evidence for no association between having a chronic disease and protein-energy malnutrition. In addition to this, strong evidence for no association was found for, among others, high number of diseases, stroke, heart failure and coronary failure [41].

TNFα is among the inflammatory markers frequently used to study processes of aging. It is primary produced by macrophages and plays a role in directing inflammatory reactions in our bodies by stimulating release of proteins, like IL-6 and CRP. TNFα is produced as an adipocytokine in adipose tissue. When adipose tissue expands to a great extent, TNFα contributes to insulin resistance [35]. Studies have demonstrated high levels of TNFα in correlation with decline in muscle mass/strength, suggesting an association with sarcopenia and malnutrition [42, 43].

The actions of TNFα are mediated by two distinct TNF receptors, receptors 1 and 2 (TNFRI and TNFRII). Our study used TNFRII level as an inflammatory marker and it was determined to be higher in patients at risk of malnutrition.

As written above, Oe et al*.* observed a significant association between TNFα and malnutrition status in older women [35]. In another study, high levels of circulating TNFα were observed in patients with Alzheimer’s disease experiencing unexplained weight loss [20]. Correia et al. further documented a significant direct correlation between higher levels of cytokines (especially TNFα), malnutrition, and decreased quality of life in patients with gastric cancer [31].

Another important biomarker is OPG, a member of the TNF receptor super-family, which we found to be associated with increased risk for malnutrition. OPG is secreted by osteoblasts, mesenchymal stem cells, fibroblasts, endothelial cells, and human adipose tissue [44]. It has pleiotropic effects over bone metabolism as well as endocrine function [45].

The role and significance of OPG seems to be broad and not fully understood. Krzanowski et al*.* postulated that OPG might be an early indicator of all-cause mortality in patients with chronic kidney disease and advanced medial arterial calcification [14]. Janda et al*.* found that elevated serum OPG levels may be useful as a prognostic marker of cardiovascular risk in dialyzed patients [15]. A higher OPG level predicts poor prognosis in subjects with heart failure, diabetes, end-stage kidney disease, coronary artery disease, acute coronary syndrome, and silent myocardial ischemia [16, 17]. Our results demonstrate association between higher level of OPG and risk of malnutrition in older persons.

In contrast to the biomarkers mentioned above, IL-18 was associated with decreased risk of malnutrition in our study. IL-18 is an inflammatory cytokine, first identified by Okamura et al. in 1995. It is found in multiple cell types including immune, hematopoietic, chondrocytic, intestinal epithelial cells, astrocytes, and microglial cells. As a member of the IL-1 family, IL-18 acts in synergy with IL-12 to activate murine and human T-cells, thereby signaling induction of inflammation, interferon-gamma production, and cell-mediated immunity [46].

IL-18, together with IL-1β, serves in activation of downstream inflammation signaling in a state of infection and injury [47, 48]. Higher levels of IL-18 have also been measured in patients with ANCA-associated vasculitis and those undergoing hemodialysis [33]. Moreover, systemic juvenile idiopathic arthritis or adult-onset Still’s disease are also characterized by high serum IL-18 concentrations and are treated by IL-18BP (binding protein).[49]. IL18 seems to be important indicator and predictor of cardiovascular death in two-year follow-up among non-diabetic patients suffering from chronic kidney disease, with a history of acute myocardial infarction in the previous year. The importance of IL-18 in the process of atherosclerotic plaque formation has been confirmed by systems analysis based on a formal model expressed in the language of Petri nets theory [50].

In addition to its role in autoimmune diseases, IL-18 contributes to the process of atherosclerosis and, therefore, by inference to aging. Clearly, reducing IL-18 activities can be viewed as possible therapeutic strategies to slow the aging process [51]. It was shown that IL-18 as a proatherogenic cytokine was associated with the development of cardiovascular disease and all-cause mortality in stable heart disease patients independent of cardiac dysfunctions [26]. It is a strong predictor of cardiovascular death in patients with stable or unstable angina [52].

Worth mentioning is fact, that one study demonstrated significantly decreased plasma IL-18 levels in patients with anorexia nervosa compared to controls. Plasma IL-18 levels directly correlated with BMI in controls, but not in patients with anorexia nervosa. These results suggest that a decline in plasma IL-18 levels in patients with anorexia nervosa is not only due to malnourishment, but other pathophysiologic changes as well. IL-18 has a role in brain’s reaction to sadness and chronic stress. Therefore, decreased levels of IL-18 may commonly occur in patients with chronic anorexia nervosa [46].

It is difficult to explain the lower IL-18 level in patients with malnutrition risk in comparison to the patients with proper nutrition. It might be a result of a relatively low number of participants. However, as an explanation, we should also take into account an appropriate treatment that causes the stability of the diseases, as evidenced by relatively good muscle mass in patients with heart failure.

An article published in November 2019 dedicated the MaNuEL Toolbox (2-year Joint Action Malnutrition in the Elderly project) shows how important is the problem of malnutrition is in older people. It was made available to effectively distribute and disseminate the MaNuEL results and recommendations, which will support researchers, healthcare professionals, policy-makers as well as educational institutes to advance their efforts in tackling the increasing problem of protein–energy malnutrition in the older population [53].

However, the authors of The MaNuEL Multicohort Meta-Analysis focused on and stressed the importance of social and physical factors contributing to malnutrition of older people. According to its results unmarried, separated, or divorced participants were more likely to develop malnutrition that married participants, whereas no association was found for widowed participants. Participants with difficulty in walking or difficulty in climbing stairs and those who were hospitalized before baseline and during follow-up had higher odds of incident malnutrition [54].

We are aware of some limitations of this study—first, the observational nature of the study does not allow to determine the causative relationship between malnutrition risk and inflammation process, this should be studied in depth later. The second, the number of patients included in this analysis is small and that limits the statistical power to detect differences between study groups, as well as do not allow for studying many possible confounders that can play role in the association between malnutrition and inflammation. Another important consideration is the lack of participants diagnosed with malnutrition, which may weaken the significance of relationships assessed. In addition to this, the group is heterogeneous and, as previously was written, relatively small, so that might influence the results. Finally, we also took into consideration that the markers are of poor specificity.

Despite these limitations, our study points out that there is a relationship between inflammation and risk of malnutrition in patients in stable period of chronic diseases. Besides, it extends a cytokine spectrum connected with unsatisfactory nutritional state.

In conclusion, the etiopathogenesis of malnutrition in older persons is complex.

Our study indicated that chronic inflammation, irrespective of the reason, plays a probable role and should be considered in evaluating nutritional status in the geriatric population; however, it also exposes an avenue where further research is needed in order to enhance our understanding and guide comprehensive patient care.
